# Genome sequence data of phytase-producing strains of *Pantoea brenneri*

**DOI:** 10.1128/mra.01032-25

**Published:** 2025-11-14

**Authors:** Aliya Suleimanova, Daria Pudova, Lidiya Sokolnikova, Elena Shagimardanova, Margarita Sharipova

**Affiliations:** 1 Laboratory of Agrobioengineering, Institute of Fundamental Medicine and Biology, Kazan (Volga Region), Federal University 473102, Kazan, Russia; 2 Loginov Moscow Clinical Scientific Center, Moscow, Russia; 3 Center for Genomics and Bioimaging, Moscow, Russia; Indiana University, Bloomington, Bloomington, Indiana, USA

**Keywords:** genome sequence, *Pantoea brenneri*, phytase

## Abstract

We present the findings from the genomes sequencing project of *Pantoea brenneri* 3.1, 3.5.1, 3.5.2, and 3.6.1 strains, sourced from the soil according to their ability to hydrolyze phytate. The genomes span 4.98 Mb for 3.1, 3.5.1, and 3.6.1 strains and 4.26 Mb for 3.5.2 strain with an average GC content of 55.9%.

## ANNOUNCEMENT

A promising solution to the steady depletion of natural mineral phosphorus reserves (1) involves utilizing phytates, a class of organic phosphorus compounds that are abundant in soil. Phytates are naturally hydrolyzed by phytases, produced by soil microorganisms (2). The described bacteria are producers of histidine acid phytases, leading to the whole-genome sequencing of *Pantoea brenneri* 3.1, 3.5.1, 3.5.2, and 3.6.1 strains (3).

Bacterial strains *P. brenneri* 3.1, 3.5.1, 3.5.2, and 3.6.1 were isolated from the rhizosphere forest soil sample (loamy sand, pH 7.12) according to their ability to hydrolyze phytate (GPS coordinates 54.833300°N, 53.000000°E). To isolate phytate-degrading bacteria, soil samples (5 g) were mixed with 45 mL of sterile tap water and shaken for 3 min on a rotary shaker. Samples were allowed to sediment, and serial 10-fold dilutions were prepared. Each dilution was plated on phytate-specific-agar plates (2% glucose, 0.4% sodium phytate, 0.2% CaCl_2_, 0.5% NH_4_NO_3_, 0.05% KCl, 0.05% MgSO_4_·7H_2_O, 0.001% FeSO_4_·7H_2_O, 0.001% MnSO_4_·H_2_O, and 3% agar, pH 7.0) (4), incubated for 5 days at 28°C and examined for halo-zones. Cultivation of *P. brenneri* strains was carried out overnight on Luria Bertani medium (10.0 g tryptone, 5.0 g yeast extract, 5.0 g NaCl, and 1000 mL water, pH 6.8–7.2) in an incubation shaking cabinet (“B. Braun,”, Germany) at 30°C and 180 rpm (3).

Genomic DNA from the strains was isolated using GeneJET Genomic DNA Purification Kit (Thermo Scientific, USA) from 5 mL of bacterial cultures. Genomic libraries were prepared using NEBNext Ultra II DNA Library Prep Kit for Illumina (New England Biolabs, USA). DNA concentration was measured with a NanoDrop 2000c Spectrophotometer (Thermo Scientific), yielding 500 ng/µL. DNA sequencing was performed on the Illumina NovaSeq 6000 platform with a 250 bp paired-end library. The quality assessment of the obtained sequencing data were assessed using the FastQC v0.12.0 program (5).

The key results of sequencing, assembly, and annotation are presented in Table 1. After read filtration with Trimmomatic v.0.32 (6) (parameters: CROP:200, SLIDINGWINDOW:4:20, ILLUMINACLIP:TruSeq3-PE:2:30:10, and MINLEN:100), 65% of the reads were retained for *de novo* genome assembly, which was performed using SPAdes_v3.15.5 (7) with the “--isolate” parameter. Key draft genome assemblies for four *Pantoea* strains and annotation metrics are provided in Table 1. The published genome of strain 3.5.1 (3) has been updated (JMRT00000000.3) with an improved assembly (higher *N*_50_, longer contigs).

**TABLE 1:** Genome assembly and gene annotation statistics of *Pantoea brenneri* strains

Strain	3.1	3.5.1	3.5.2	3.6.1
Total length, Mb	4.98	4.98	4.26	4.98
Number of contigs	29	31	24	31
Number of scaffolds	27	29	22	28
Contig *N*_50_	606.3 kb	562.4 kb	741.1 kb	562.5 kb
Scaffold *N*_50_	606.3 kb	707.1 kb	2.3 Mb	606.2 kb
GC content, %	56	56	56	56
Coverage	56.0×	145.0×	89.0×	63.0×
Number of CDSs	4,509	4,508	3,863	4,509
Number of rRNAs	20	27	20	22
Number of tRNAs	77	77	74	77
Number of raw reads	5 M	25.4 M	11.9 M	5.2 M
Accession for raw reads	SRR31360111	SRR31370902	SRR31360620	SRR31363392
Assembly	JBCGBG000000000.1	JMRT00000000.3	JBCGBH000000000.1	JBCGBI000000000.1

List of phytase encoding sequences in *P. brenneri* genomes				
Esterase-like activity of phytase family protein (WP_031374876.1)	NZ_JBCGBG010000002.1 488171-489523 (+)	NZ_JMRT03000002.1 488301-489653 (+)	Absent	NZ_JBCGBI010000002.1 168907-170259 (+)
Bifunctional glucose-1-phosphatase/inositol phosphatase (WP_031376103.1)	NZ_JBCGBG010000001.1 1646702-1648429 (−)	NZ_JMRT03000001.1 1646423-1648150 (−)	NZ_JBCGBH010000001.1 1646650-1648377 (−)	NZ_JBCGBI010000001.1 1646932-1648659 (-)

The phylogenetic position of the strains was established using MLSA analysis based on the partial sequences of *fusA*, *pyrG*, *leuS*, *gyrB*, and *rpoB* genes. Phylogenetic analysis was performed using the Neighbor-Joining method with a bootstrap value of 1,000 iterations in the MEGA v11.0 program (8) with default parameters. The studied isolates formed a separate cluster with *P. brenneri* species.

The genomes were annotated using the NCBI Prokaryotic Genomes Annotation Pipeline (9) with default parameters (Table 1). Three of the four strains contained two genes encoding phytase. Strain 3.5.2 was lacking esterase-like activity of phytase family (Table 1).

In conclusion, this study undertook the genomic sequencing of four rhizospheric strains of *P. brenneri* to unravel the genetic background of their potential biotechnological application as a PGP agent.

## Data Availability

Data of whole-genome shotgun sequencing project uploaded to DDBJ/EMBL/GenBank databases under accession numbers JBCGBG000000000.1, JMRT00000000.3, JBCGBH000000000.1, and JBCGBI000000000.1 for *P. brenneri* 3.1, 3.5.1, 3.5.2, and 3.6.1, respectively. BioProject accession numbers for *P. brenneri* 3.1 (PRJNA1095726), 3.5.1 (PRJNA246264), 3.5.2 (PRJNA1095731), and 3.6.1 (PRJNA1095733), and BioSample accession numbers for *P. brenneri* 3.1 (SAMN40733421), 3.5.1 (SAMN02746163), 3.5.2 (SAMN40733431), and 3.6.1 (SAMN40733516). Accession numbers for the raw reads for *P. brenneri* 3.1 (SRR31360111), 3.5.1 (SRR31370902), 3.5.2 (SRR31360620), and 3.6.1 (SRR31363392).
